# Biopolymer Development from Agro-Food and Aquaculture By-Products with Antioxidant Hydrolysates of *Cyprinus carpio*, Produced via Enzymatic Preparations of Pineapple and Papaya

**DOI:** 10.3390/ijms27010148

**Published:** 2025-12-23

**Authors:** Guadalupe López-García, Octavio Dublán-García, Francisco Antonio López-Medina, Ana Gabriela Morachis-Valdez, Karinne Saucedo-Vence, Daniel Arizmendi-Cotero, Daniel Díaz-Bandera, Gerardo Heredia-García, Angel Santillán-Álvarez, Luis Alberto Cira-Chávez, Baciliza Quintero-Salazar

**Affiliations:** 1 Laboratorio de Alimentos y Toxicología Ambiental, Facultad de Química, Universidad Autónoma del Estado de México, Carr. Toluca-Ixtlahuaca Km. 15, El Cerrillo Piedras Blancas, Toluca 50200, Mexico; logg_sf@hotmail.com (G.L.-G.); flopezmedina92@gmail.com (F.A.L.-M.); agmorachis@gmail.com (A.G.M.-V.); karinne.saucedo@utvtol.edu.mx (K.S.-V.); arcoda21@gmail.com (D.A.-C.); ddiazb@uaemex.mx (D.D.-B.); gherediag516@alumno.uaemex.mx (G.H.-G.); 2 Dirección de Procesos Alimentarios y Química en Alimentos, Área de Biotecnología, Unidad Académica de Capulhuac, Universidad Tecnológica del Valle de Toluca (UTVT), Calle s/n, 611 Oriente de Colonia, Lomas de San Juan, Municipio, Capulhuac de Mirafuentes 52700, Mexico; 3 División de Gastronomía, Tecnológico Nacional de México (TecNM), Tecnológico de Estudios Superiores de Valle de Bravo, Km. 30 de la Carretera Nacional Federal Monumento-Valle de Bravo, Ejido de San Antonio de La Laguna, Valle de Bravo 51200, Mexico; angel.sa@vbravo.tecnm.mx; 4 Departamento de Biotecnología y Ciencias Alimentarias, Instituto Tecnológico de Sonora, Calle 5 de Febrero 818 Sur, Centro, Urb. No. 1, Cd Obregón 85000, Mexico; luis.cira@itson.edu.mx; 5 Facultad de Turismo y Gastronomía, Campus “El Rosedal”, Universidad Autónoma del Estado de México, Carretera Toluca-Atlacomulco Km. 14.5, Toluca 50200, Mexico; bquinteros@uaemex.mx

**Keywords:** biopolymer, protein hydrolysates, *Cyprinus carpio*, enzymatic hydrolysis, antioxidant activity, circular economy

## Abstract

This study describes the development of a biodegradable biopolymer formulated from protein–polysaccharide matrices enriched with antioxidant hydrolysates obtained from *Cyprinus carpio* by-products. The hydrolysates were produced through targeted enzymatic hydrolysis using plant-derived proteases, yielding peptide fractions with relevant radical-scavenging activity. Molecular characterization (DSC) confirmed the presence of thermal stability suitable for cold-chain applications, while the resulting biopolymer displayed flexible and cohesive structural behavior. The material was evaluated as an edible coating for raspberries stored at 4 °C. Coatings containing the hydrolysates, particularly those generated with bromelain, more effectively slowed physicochemical deterioration, modulated oxidative reactions, and helped to preserve nutritional quality during storage. These findings indicate that integrating bioactive peptide hydrolysates into biodegradable polymer networks enhances their functional performance, offering a sustainable approach for food preservation and valorization of agro-aquaculture residues.

## 1. Introduction

Food loss and waste pose significant environmental and economic challenges. Approximately one-third of food intended for human consumption is never eaten, contributing to greenhouse gas emissions, water depletion, and inefficient land use [1]. Losses are particularly high in fruits and vegetables (≈45%) and fisheries (>35%), sectors that generate substantial organic residues rich in proteins and structural polysaccharides [2]. In Mexico, citrus processing, dairy operations, and aquaculture production yield large amounts of underutilized by-products that contribute to environmental pollution when improperly managed [3,4]. Valorization strategies that convert these residues into functional biomolecules align with circular bioeconomy principles and offer sustainable alternatives for waste reduction.

Enzymatic hydrolysis has emerged as a key method for transforming protein-rich residues into bioactive peptides with antioxidant, antimicrobial, and functional properties. Common carp (*Cyprinus carpio*), widely produced in Mexico, yields significant by-products that constitute an excellent substrate for hydrolysis [5,6]. Papain and bromelain, plant-derived cysteine proteases, demonstrate broad substrate specificity and cleave peptide bonds at sites enriched in hydrophobic, aromatic, or basic amino acids, releasing low-molecular-weight peptides (<3 kDa) with strong bioactivity [7,8,9,10]. These peptides often contain Tyr, Trp, Phe, Pro, and sulfur-containing residues, which confer radical-scavenging capacity, hydrogen atom transfer ability, and metal-chelating potential [11,12,13]. Hydrolysates derived from fish by-products typically exhibit enhanced solubility and improved antioxidant performance due to their collagen- and myofibrillar-derived sequence motifs [14,15].

Mechanistically, the antioxidant effects of these peptides arise from their ability to donate protons or electrons to stabilize reactive oxygen species, or to chelate pro-oxidant metal ions that catalyze lipid oxidation [11,12,13]. Papain and bromelain hydrolysates from tilapia, catfish, and marine species have demonstrated strong DPPH (2,2-Diphenyl-1-picrylhydrazyl) and ABTS (2,2′-azino-bis(3-ethylbenzthiazoline sulfonic acid)), radical-scavenging activity, ACE-inhibitory potential (angiotensin-converting enzyme, ACE), and functional properties relevant to food preservation [16,17]. Their high solubility and interfacial reactivity also facilitate their integration into biopolymer matrices, where they can interact through hydrogen bonding, electrostatic attraction, and hydrophobic associations, influencing polymer morphology and functional performance [18,19,20,21].

Biopolymers derived from polysaccharides and proteins can function as effective carriers for bioactive peptides, enabling controlled interactions with food surfaces and providing oxidative protection. Pectin-rich agro-residues—such as citrus peel—have been shown to form cohesive, biodegradable films with favourable mechanical and barrier properties, supporting their use as matrices for functional edible coatings [22]. The incorporation of peptide-rich hydrolysates can further modify polymer networks through hydrogen bonding and hydrophobic interactions, enhancing flexibility, stability, and antioxidant functionality within the matrix. Edible coatings enriched with bioactive peptides have demonstrated the capacity to delay senescence and reduce oxidative degradation in fresh fruits, thereby preserving key physicochemical quality attributes during storage [18,19,20,21].

Based on this rationale, the present study aimed to valorize aquaculture and citrus by-products by developing a biodegradable biopolymer composed of orange peel fiber, fish gelatin, whey, and glycerol, enriched with peptide-rich hydrolysates derived from *Cyprinus carpio* by-products. Specifically, the research investigates the molecular characteristics and antioxidant potential of papain- and bromelain-derived hydrolysates; their integration into the biopolymer matrix; and their functional performance as edible coatings for raspberries. By linking peptide structure–function relationships with biopolymer performance, this work contributes to the design of sustainable preservation systems aligned with circular food economy principles.

Building on these advances, the present research expands current knowledge by employing two plant-derived proteases (papain and bromelain) to generate differentiated peptide profiles from *Cyprinus carpio* by-products and by characterizing their structural and functional attributes using a multi-technique approach that includes FTIR (Fourier-transform infrared spectroscopy), DSC (differential scanning calorimetry), and in silico analysis. The incorporation of these peptide-rich hydrolysates into a biodegradable biopolymer matrix composed of citrus fiber, fish gelatin, and whey provides a robust framework for assessing how enzyme-specific fragmentation patterns influence polymer interactions and antioxidant performance. Applying this system to raspberries, one of the most perishable fruits, further enhances our understanding of the relationship between peptide structure, biopolymer behavior, and postharvest quality preservation, thereby broadening the technological potential of peptide-enriched edible coatings within circular bioeconomy strategies.

Taken together, these considerations led us to integrate the molecular principles of peptide generation, biopolymer assembly, and antioxidant functionality to develop a biodegradable coating enriched with papain, and bromelain-derived hydrolysates. By examining the structure, function relationships of the resulting peptides and their interactions within the biopolymer matrix, this work elucidates how these bioactive components contribute to oxidative stability and quality preservation in fresh raspberries, providing a mechanistic basis for sustainable food preservation systems.

## 2. Results

### 2.1. Electrophoretic Profile of Enzymatic Preparations

**Figure 1:**
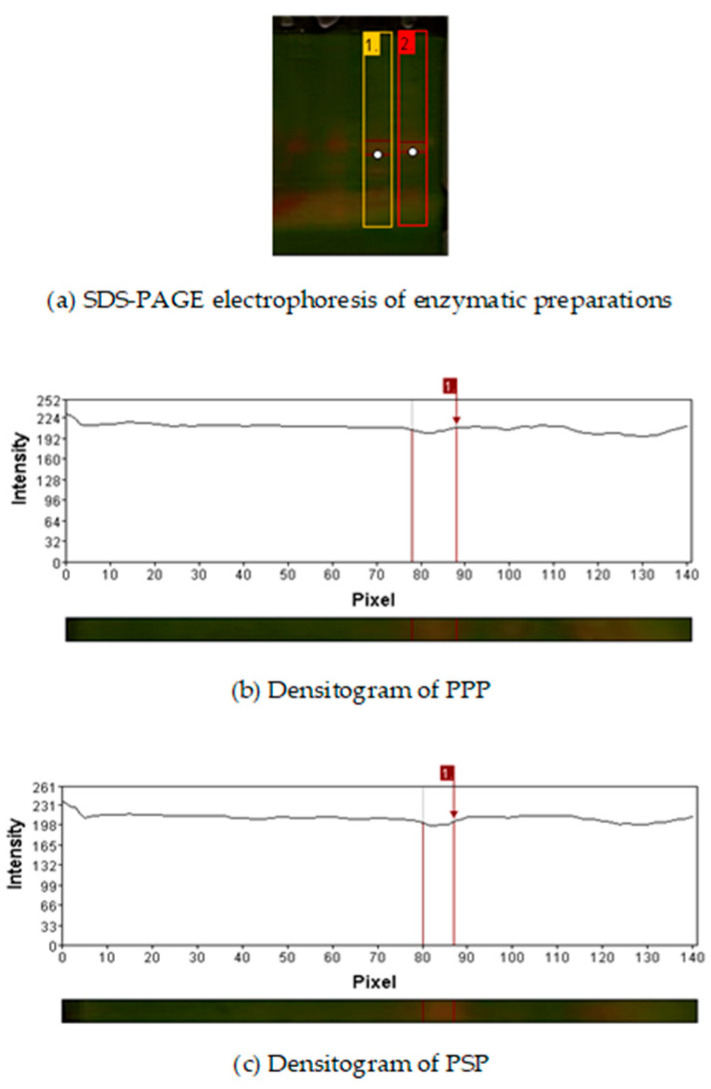
(**a**) SDS-PAGE electrophoresis of enzymatic preparations. Lane 1 corresponds to the pineapple peel enzymatic preparation (PPP), and lane 2 corresponds to the papaya seed enzymatic preparation (PSP). White dots indicate the protein bands selected for densitometric analysis. (**b**) Densitogram of PPP. (**c**) Densitogram of PSP.

Figure 1 shows the SDS-PAGE of the PPP and PSP. It was observed that extraction methods do not have a significant effect over enzymatic preparations, since same electrophoretic profile was observed. Retention factors of the samples were 0.462 for PPP and 0.542 PSP. Their approximate molecular weights (24 kDa for the pineapple peel enzymatic preparations and 21 kDa for the enzymatic preparations of papaya seed) were determined. These values correspond to those reported for bromelain and papain [23,24].

### 2.2. Proteolytic Activity of the Enzymatic Preparations

In order to quantify PP and PS to be added to fish flour for enzymatic hydrolysis, proteolytic activity was determined (Table 1), specific activity between enzymatic preparations and commercial pepsin, were compared as positive control. It was observed that PPP presented the highest specific activity in contrast to CP, and PSP presented the lowest; this difference is statistically significant (*p* < 0.05). The lower activity of PSP could be due to the extraction process of each enzymatic preparation while the PSP was dried seed the PPP was a buffer extract of pineapple peels, as well as PSP could contain a mixture of enzymes such as papain, chymopapain and lysozyme [24,25].

### 2.3. Hydrolysates

#### 2.3.1. FTIR Analysis

**Figure 2:**
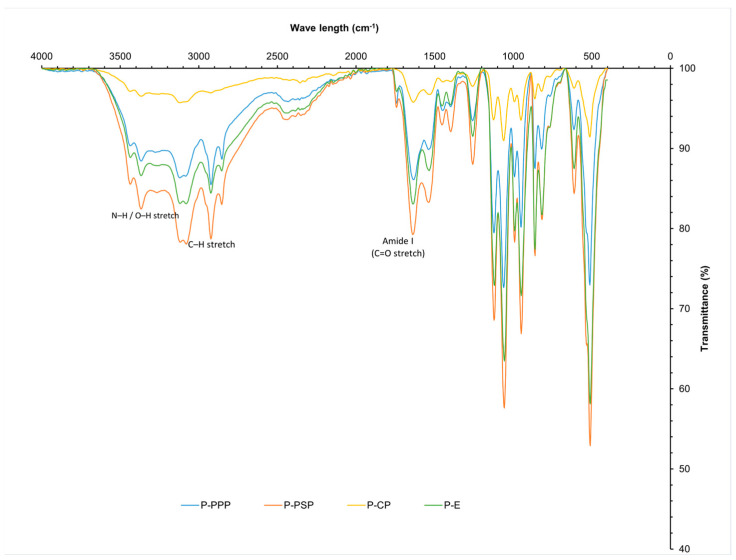
Infrared spectrum of protein hydrolysates prepared from common carp (*Cyprinus carpio*) waste.

The FTIR spectra of the carp hydrolysates (Figure 2) exhibited characteristic peptide-bond signals, including broad N–H stretching bands at 3430–3367 cm^−1^ (primary amines) and prominent Amide I and Amide II bands at 1637 and 1535 cm^−1^, corresponding to C=O stretching and N–H bending/C–N stretching, respectively. Additional vibrations in the 1230–1030 cm^−1^ region were attributed to C–N and C–C stretching of the peptide backbone. These spectral assignments are consistent with the typical vibrational modes reported for protein and peptide structures in infrared spectroscopy [26].

Because absolute peak intensities may vary with sample loading, comparisons among enzymatic treatments were based on relative spectral behaviour, including proportional attenuation of the amide bands, changes in band shape, and formation of shoulders, rather than on absolute absorbance values. Under this criterion, PPP displayed the most pronounced modification of the Amide I and II regions, showing greater proportional attenuation and clearer band narrowing features associated with extensive peptide-bond cleavage and the release of low-molecular-weight, hydrophobic peptides, consistent with the known proteolytic profile of bromelain toward fish proteins [27,28].

PSP showed an intermediate pattern, exhibiting moderate proportional decreases in the amide regions together with subtle band-shape alterations. In contrast, the pepsin hydrolysate (P-CP) presented only slight shifts and broadening of the Amide I band, suggesting conformational rearrangement with limited chain fragmentation. Overall, these relative spectral changes support that all enzymatic treatments induced protein hydrolysis, with PPP generating the most extensively modified peptide population.

#### 2.3.2. In Silico Analysis

In silico screening was performed to identify antioxidant peptide motifs that could plausibly arise from the fragmentation patterns observed in the hydrolysates. Searches in the BaAMPs and BIOPEP-UWM databases revealed the presence of the Ala–Tyr (AY) dipeptide previously annotated in *Cyprinus carpio* proteins. AY is a well-characterized antioxidant motif whose activity is attributed to the phenolic side chain of tyrosine, enabling hydrogen-donation, radical-quenching, and π-electron delocalization, combined with alanine-related conformational flexibility [29].

Because database-derived predictions only reflect peptides previously reported for carp proteins, AY should be interpreted as representative of the type of small, low-molecular-weight fragments expected under extensive hydrolysis rather than an exhaustive peptide profile. Its identification aligns with the FTIR-derived evidence for PPP, which showed the strongest amide-band depletion, a pattern typically associated with the release of hydrophobic and aromatic peptides with antioxidant potential. Thus, the in silico results support the molecular interpretation obtained from structural analyses.

#### 2.3.3. Thermal Stability (DSC) and Molecular Relationship with FTIR

**Figure 3:**
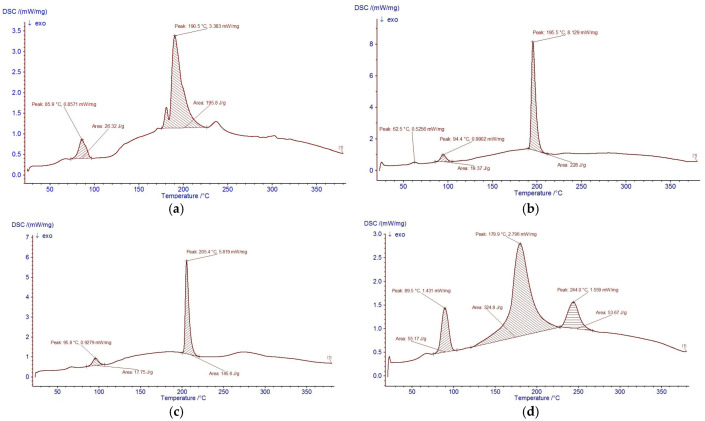
Thermal stability of (**a**) P-E, (**b**) P+CP, (**c**) P+PPP, and (**d**) P+PSP, as determined by differential scanning calorimetry (DSC). Arrows indicate the direction of the exothermic heat flow and highlight the main thermal transition peaks observed in each sample.

The thermal behaviour of the hydrolysates was analysed by DSC (Figure 3). All samples exhibited an initial endothermic transition between 85–100 °C, associated with the loss of free and weakly bound water. This event corresponds to the broad O–H and N–H stretching regions (3000–3600 cm^−1^) observed in FTIR, indicating that the hydrolysates maintain structural stability up to approximately 90 °C.

At higher temperatures, the hydrolysates displayed one or more intense transitions attributed to the thermal degradation of peptide structures. PPP showed a major transition at 205.4 °C, consistent with its extensive hydrolysis and the presence of shorter, more thermally labile peptide chains. PSP exhibited transitions at 179.9 and 244.0 °C, reflecting a heterogeneous population of peptides with varying thermal stability. Pepsin-derived hydrolysates (P-CP) and the non-enzymatic hydrolysate (P-E) showed fewer and weaker transitions, consistent with a greater presence of intact or partially hydrolysed proteins [30,31,32,33].

Integrating FTIR and DSC findings, the hydrolysis–fragmentation pattern follows the order PPP > PSP > CP > P-E, where greater amide-band depletion correlates with earlier and sharper high-temperature transitions, reflecting smaller, more mobile peptide chains. These structural differences help explain the antioxidant performance of the hydrolysates and support their suitability for incorporation into biopolymer matrices intended for refrigerated or mild-temperature food preservation.

In silico analysis was performed to identify antioxidant peptide motifs that could plausibly arise from the enzymatic fragmentation patterns observed in the hydrolysates. Searches in the BaAMPs and BIOPEP-UWM databases highlighted the presence of the Ala–Tyr (AY) dipeptide in annotated *Cyprinus carpio* sequences. AY is a well-characterized antioxidant motif, whose activity derives from the phenolic side chain of tyrosine, capable of hydrogen donation, radical quenching, and stabilization through π-electron delocalization, combined with the conformational flexibility provided by alanine [34].

Because database-driven prediction reflects only peptides previously reported in carp proteins, the recovered AY sequence should be viewed as an indicator of the type of low-molecular-weight fragments expected under extensive hydrolysis conditions rather than a complete list of all peptides formed. Its detection is consistent with the FTIR-derived evidence for PPP, which exhibited the strongest amide-band depletion, a pattern typically associated with the release of small hydrophobic and aromatic peptides exhibiting antioxidant potential. Thus, the in silico data support the molecular interpretation suggested by the structural analyses.

#### 2.3.4. Thermal Stability of Carp Hydrolysates and Molecular Relationship with FTIR

The thermal behaviour of the hydrolysates was assessed by DSC (Figure 3). All samples exhibited an initial endothermic transition between 85–100 °C, corresponding to the loss of free and weakly bound water. This event correlates with the broad O–H and N–H stretching bands (3000–3600 cm^−1^) observed in FTIR and indicates that the hydrolysates are structurally stable up to ~90 °C, a temperature range consistent with mild food processing and coating applications [35,36,37,38].

At higher temperatures, the hydrolysates displayed one or more intense transitions associated with the thermal degradation of peptide structures. The non-enzymatic hydrolysate (P-E) showed a single broad transition centred at 190.5 °C, characteristic of cooperative degradation of relatively intact proteins and associated lipids. In contrast, the enzymatically produced hydrolysates exhibited peak splitting and shifts in the main transition temperatures. PPP presented a sharp transition at 205.4 °C, whereas PSP showed two major events at 179.9 and 244.0 °C, and P-CP displayed an intermediate transition at 195.5 °C. These differences reflect changes in molecular weight distribution and chain mobility: hydrolysis partially disrupts the native protein network, decreases cooperative interactions, and generates shorter peptide chains that denature or decompose over broader and, in some cases, lower temperature ranges [35,36,37,38,39,40].

Hydrolysates obtained with enzymatic treatments (PPP, PSP, CP) showed broader and more pronounced high-temperature transitions than P-E, indicating the presence of heterogeneous populations of low-molecular-weight peptides with different thermal stabilities. PPP and PSP, in particular, displayed sharper high-temperature peaks and greater enthalpy changes, consistent with extensive fragmentation into short peptides that undergo ordered structural rearrangements before degradation. Similar DSC patterns, where increasing degree of hydrolysis shifts denaturation/decomposition peaks and modifies transition enthalpies, have been reported for fish and legume protein hydrolysates, and have been attributed to reduced molecular size and altered hydrophobic interactions within the peptide matrix [39,40].

The combined FTIR and DSC analyses therefore support a hydrolysis pattern that follows the order PPP > PSP > CP > P-E. Greater depletion of amide bands in FTIR correlates with earlier and more defined high-temperature transitions in DSC, reflecting smaller, more mobile peptide chains. These structural modifications help explain the antioxidant performance observed for the hydrolysates and support their incorporation into biopolymer matrices intended for refrigerated or mild-temperature food preservation.

#### 2.3.5. Soluble Protein Content of Carp Protein Hydrolysates

The protein content of the carp hydrolysates obtained with different enzymatic preparations showed statistically significant differences between the hydrolysates (*p* < 0.05) and the common carp protein (Table 2). P-PSP presented a lower protein content, even when enzymatic preparations with the same proteolytic activity were used. However, they are also the preparations with the highest antioxidant activity, which indicates that the type of protease had an effect in the production of peptides, due to the specificity of the enzymes which these contain, and P-PSP were able to generate peptide sequences with a higher content of hydrogen or electron donors amino acids [41,42].

#### 2.3.6. Antioxidant Activity (ABTS^•+^ and DPPH^•^ Inhibition)

The antioxidant responses of the hydrolysates differed significantly among enzymatic treatments (Table 3). P-PSP exhibited the highest radical-scavenging activity in both DPPH^•^ and ABTS*^•+^* assays. This effect can be mechanistically explained by the known cleavage specificity of papain, which preferentially hydrolyzes peptide bonds adjacent to Lys, Arg, and hydrophobic residues, generating short peptides enriched in basic and aromatic amino acids (e.g., Tyr, Phe, Trp) [10,43,44]. Peptides containing these residues exhibit strong electron-donating and hydrogen-atom transfer capacities due to their phenolic or indole side chains, which promote resonance stabilization of radical intermediates [45].

Furthermore, papain hydrolysates from fish typically produce peptides <3 kDa with higher surface hydrophobicity and greater exposure of radical-reactive residues, enhancing their accessibility to free radicals [46]. Increased hydrophobicity is strongly correlated with DPPH^•^ scavenging efficiency, because DPPH^•^ is a hydrophobic radical measured in organic media; therefore, peptides with greater non-polar character interact more effectively with the radical species [47]. This explains why P-PSP exhibited the highest DPPH^•^ inhibition among samples.

In contrast, the ABTS^•+^ assay is performed in aqueous medium and responds not only to hydrophobic peptides but also to small, charged sequences with high electron-transfer capacity. The strong performance of P-PSP in ABTS^•+^ suggests that papain generated peptides with favorable redox-active residues (Tyr, Trp, His, Met) and adequate solubility, enabling efficient electron transfer in aqueous environments [45,48].

PPP showed intermediate antioxidant activity, consistent with bromelain’s broader but less selective cleavage pattern. Bromelain tends to release peptides with diverse lengths and lower aromatic enrichment compared with papain [49]. This structural variability may reduce the density of potent radical-scavenging motifs, explaining the lower activity compared with P-PSP. CP exhibited the lowest antioxidant response, consistent with the FTIR-based inference of partial hydrolysis and the presence of longer peptide fragments with fewer exposed reactive residues.

Overall, the structure–activity relationship indicates that the superior antioxidant capacity of P-PSP arises from its higher proportion of short, hydrophobic, and aromatic-rich peptides—properties that enhance both H-atom transfer (DPPH^•^ mechanism) and single electron transfer (ABTS^•+^ mechanism). These mechanistic trends align with peptide behavior reported in other papain-hydrolyzed fish proteins, including tilapia, mackerel, and catfish [43,46,48].

#### 2.3.7. Reducing Power of Carp Protein Hydrolysates

**Figure 4:**
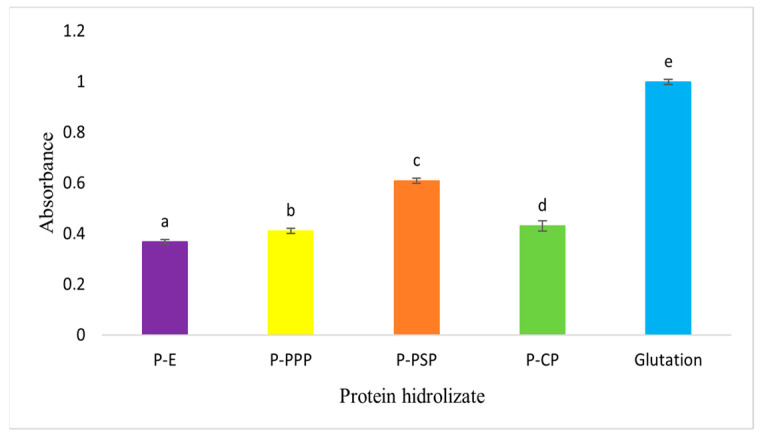
Reducing power of protein hydrolysates prepared from common carp (*Cyprinus carpio*) waste. Different letters indicate statistically significant differences.

In Figure 4, it is shown that the carp waste hydrolysates obtained with enzymatic preparations of papaya seed are those which exhibited a greater reducing power and that the hydrolysates that were obtained with other enzymatic preparations show a statistically significant difference (*p* < 0.05), about those of the papaya seed. The carp control also presented antioxidant activity and is the one that exhibited the lowest reducing power. According to Aryanti et al. [50], samples with the highest reducing power are the best electron donors; thus, the results of this study show that the hydrolysates of the common carp subproducts obtained with papaya and pineapple enzymatic preparations have a greater reducing power than the hydrolysates obtained with commercial pepsin (*p* < 0.05). Our results show that the common carp hydrolysates have a greater reducing power than the sepia hydrolysates obtained with chymotrypsin and trypsin, as reported by Ktari et al. [51]. In both studies, it was observed that the different enzymatic preparations, because of the specificity of each of the enzymes that they are conformed by, generate hydrolysates with different capacities to donate electrons to free radicals and these peptides could therefore be employed to prevent lipidic peroxidation catalyzed by metals such as Fe^2+^ and Cu^2+^ [52,53].

The choice of enzymatic preparation is a determining factor in reducing power of carp waste hydrolysates. Papaya and pineapple seed enzyme preparations produce hydrolysates with higher antioxidant capacity, suggesting their potential application in preventing metal-induced lipid peroxidation.

Compared with the glutathione standard (GSH), all enzymatic hydrolysates showed lower absolute reducing power values, as expected for peptide mixtures versus a pure tripeptide with high redox capacity. However, P+PSP approached GSH performance more closely, exhibiting approximately 60% of the control’s absorbance at 700 nm. P+PPP and P+CP displayed intermediate reducing power (40% and 38%, respectively), whereas P-E showed the lowest activity (35%), consistent with its limited hydrolysis. This ranking confirms that papain- and bromelain-derived peptides serve as effective electron donors, although not as potent as glutathione, aligning with structure–function relationships reported for fish protein hydrolysates [50,51].

### 2.4. Characterization of Raw Materials for Biopolymer Production

#### 2.4.1. Orange Fiber

**Figure 5:**
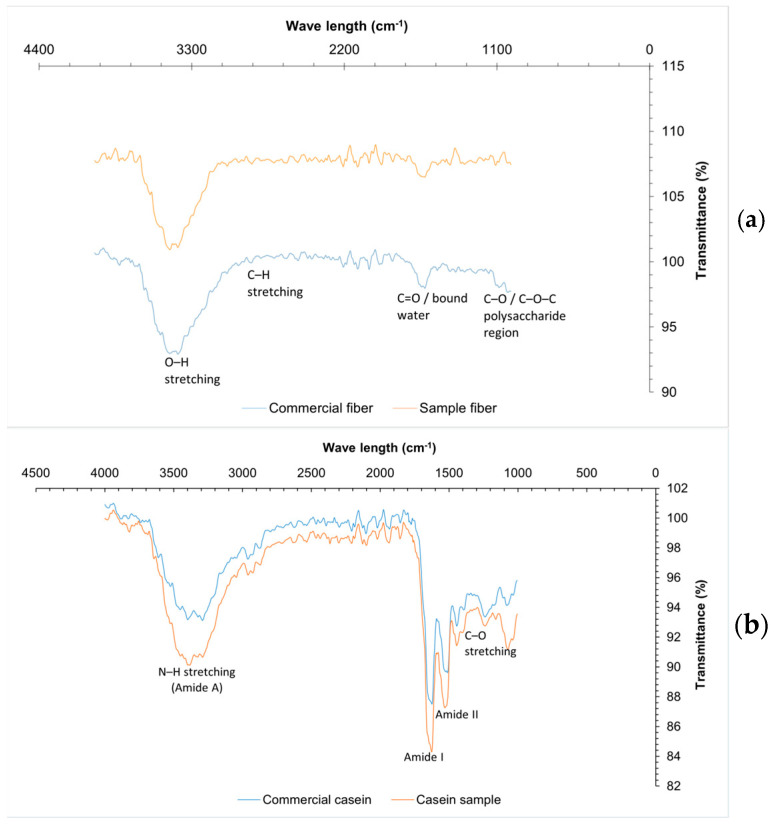

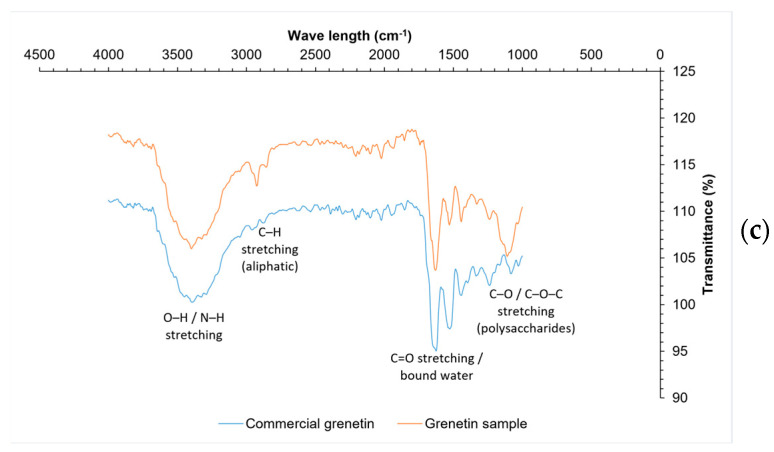
FTIR from materials for biopolymer production: (**a**) fiber, (**b**) casein, (**c**) grenetin.

Table 3 shows the composition of orange fiber. [54] reported a moisture value of 4.5% for citrus fiber, this value is higher than that of the fiber obtained; this value should be ˂10% to avoid the proliferation of microorganisms and to be able to prolong the useful life of the product. As for ash content, the value obtained (3.54%) indicates a high content of minerals such as calcium, phosphorus and magnesium, components found in orange peel flour, according to Rincón et al. [55]. The FTIR spectra (Figure 5a) of the commercial and sample fibers reveal the presence of characteristic functional groups associated with polysaccharide structures. Both samples exhibit a broad absorption band around 3300 cm^−1^ corresponding to O–H stretching, though it is more intense in the commercial fiber, indicating a higher content of hydroxyl groups, likely due to greater cellulose or hemicellulose content and moisture retention. The band near 2900 cm^−1^, related to C–H stretching in aliphatic chains, is also more pronounced in the commercial fiber. Additionally, the absorption around 1650 cm^−1^, associated with C=O groups or bound water, appears stronger in the commercial fiber, suggesting higher structural water or carbonyl compound content. Both spectra show signals between 1000 and 1200 cm^−1^ corresponding to C–O and C–O–C vibrations, typical of ether and polysaccharide linkages. Overall, commercial fiber shows higher transmittance intensities, indicating a more abundant or pure composition of structural polysaccharides, while the sample fiber, although similar in functional groups, appears to contain lower concentrations or a less refined structure [56,57].

**Table 3:** Proximal composition and quality specifications of raw materials used in biopolymer formulation.

Parameter	Orange Fiber	Casein	Grenetin	Specification Range
Moisture (%)	3.61 ± 0.2	4.0 ± 0.1	5.9 ± 0.2	1.0–12.5 [58,59]
Ashes (%)	3.54 ± 0.1	4.0 ± 0.5	1.6 ± 0.4	<15.0 [58,59]
Lipids (%)	0.60 ± 0.1	32.0 ± 3.5	—	26.0–42.0 [58,59]
pH	—	—	4.6 ± 0.1	≥4.0 [60,61]
Transmittance (%)	—	—	1.1 ± 0.1	By agreement [60,61]
Viscosity (cps)	—	—	29.0 ± 0.8	30 [60,61]
Gel strength (N)	—	—	5.0 ± 0.4	1.5–5.8 [60,61]

#### 2.4.2. Casein

The results for the composition of whey protein (casein) are within the specifications established in NOM-243-SSA1-2010 [62] (Table 4), coinciding with that reported by Zhao et al. [63]. The FTIR spectra (Figure 5b) of the commercial and sample casein reveal distinct differences in the intensity of key functional groups associated with protein structures. Both spectra exhibit broad absorption bands between 3430 cm^−1^ and 3367 cm^−1^, corresponding to the asymmetric and symmetric stretching vibrations of N–H bonds, which are more intense in the commercial casein, indicating a higher protein concentration or greater molecular order. The strong band around 1650 cm^−1^, attributed to the C=O and C–N stretching vibrations of the Amide I group, and the band at approximately 1565 cm^−1^, associated with N–H bending and C–N stretching (Amide II), are also more pronounced in the commercial sample. These differences suggest a higher degree of protein integrity and purity in the commercial casein compared to the sample, which exhibits slightly lower transmittance and less intense absorption across these regions. Despite these variations, both spectra confirm the presence of characteristic protein functional groups, validating the proteinaceous nature of the sample casein and its compositional similarity to the commercial reference [64,65].

#### 2.4.3. Grenetin

The grenetin obtained is within the specifications established in [66], coinciding with that reported by Avena-Bustillos et al. [61], having a gel strength of 187° Bloom. The FTIR spectra (Figure 5c) of the commercial and sample gelatin reveal characteristic functional groups associated with protein structures. Both samples exhibit a broad absorption band around 3300 cm^−1^, corresponding to O–H and N–H stretching, though the commercial gelatin shows greater intensity, suggesting a higher degree of hydrogen bonding or bound water content. The Amide I band (~1650 cm^−1^), related to C=O stretching, and the Amide II band (~1550 cm^−1^), associated with N–H bending and C–N stretching, are more pronounced in the commercial sample, indicating a higher protein content and more defined secondary structure. Additionally, the band near 1390 cm^−1^, assigned to C–O stretching or carboxyl group vibrations, appears with greater intensity in the commercial gelatin. These differences suggest that the commercial gelatin possesses a more ordered molecular structure or higher purity compared to the sample gelatin. Nonetheless, the presence of similar functional groups in both spectra confirms their proteinaceous nature and the gelatin-specific molecular features [67,68].

The formation of a polymeric matrix between orange fiber, casein, and gelatin can be explained by the presence of specific functional groups identified through FTIR spectroscopy. The absorption bands corresponding to O–H, C=O, N–H, and C–N bonds indicate that these biomolecules can interact via hydrogen bonding, electrostatic interactions, and hydrophobic forces. These interactions enable the formation of a stable structure between polysaccharides and proteins, as reported in recent studies where the combination of polysaccharides with soy or whey proteins modified the texture, digestibility, and functionality of biopolymeric systems [69,70,71]. These findings confirm the importance of spectroscopic characterization for optimizing technological applications in food and biomaterial development.

### 2.5. Biopolymer Based on Agrifood Byproducts

#### Biopolymer Characterization

**Figure 6:**
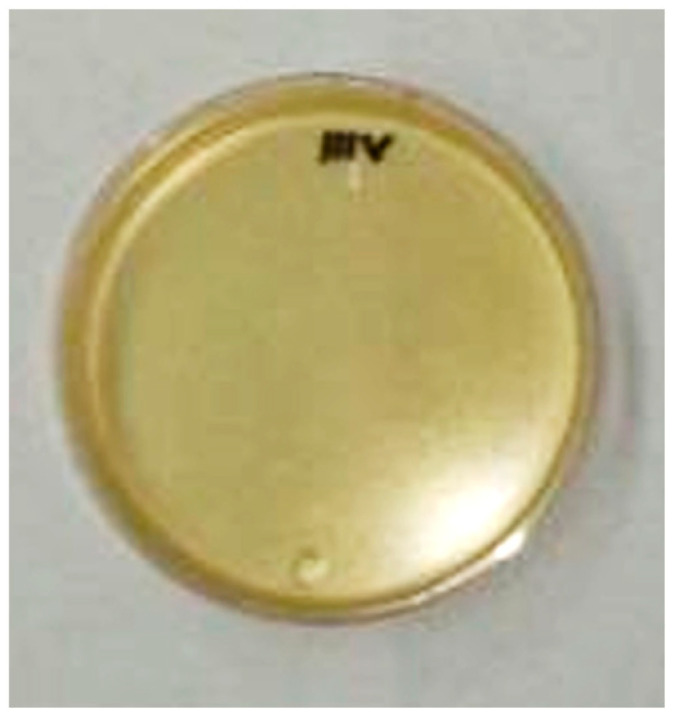
Biopolymer formulated with whey, grenetin, citrus peel fiber, and glycerol (This figure shows only the final optimized formulation; comparative analyses were conducted previously in undergraduate theses from the Food Chemistry program at UAEMex).

The biopolymer obtained from the casein–grenetin–citrus peel fiber dispersion, using glycerol as plasticizer, exhibited a smooth and thin structure with homogeneous surfaces and a slight yellow coloration (Figure 6). This figure corresponds exclusively to the final optimized formulation, as the comparative evaluation of alternative compositions was previously carried out in undergraduate theses from the Food Chemistry program at UAEMex [58,70,71].

### 2.6. Physical Properties

Table 5 presents the mean values of the physical properties of the biopolymer film. These values correspond exclusively to the single optimized formulation used in this study, which was previously established in undergraduate theses from the Food Chemistry program at UAEMex. No comparative formulations were produced here; therefore, the reported values reflect the mean ± standard deviation of triplicate measurements taken from the same biopolymer matrix.

The film exhibited a moisture content of 33.80 ± 1.24%. According to Du et al. [72], interactions between grenetin and other components in the matrix may influence this parameter. In addition, the 9% glycerol concentration contributed to the elevated moisture content, consistent with Cazón et al. [73], who reported that higher plasticizer concentrations significantly increase moisture due to the hygroscopic nature of glycerol [70,71].

Water holding capacity (WRC) is related to the ability to bind water in the film matrix [69], reported a higher moisture loss (29.4%) at low whey protein concentration when testing different concentrations of whey protein isolate (0.4 and 0.8%) in alginate-based gels. In developed biopolymer, the addition of whey protein increased the amount of water bound in the networks that form the film, since it contains hydrophilic groups that due to the effect of the thermal process interact with water, increasing the WRC.

Solubility showed the ability of the films to dissolve in water, so when ingested it can be adequately digested or, if released into the environment, it can decompose naturally, the range of this property was found to be 49.78 ± 4.75 as shown in Table 4. This may be due to the fact that, in the case of fiber, its composition contains a higher percentage of insoluble fiber.

The film-forming solution exhibited a viscosity that falls within the range commonly reported for protein–polysaccharide dispersions (Table 4), as documented by Herrera-Vázquez et al. [74]. This viscosity ensured adequate coating and uniform surface coverage during the dipping process.

Colorimetric analysis showed high luminosity (L*) values, similar to those reported by Galus et al. [19] for whey protein–based films. The a* coordinate exhibited negative values, indicating a greenish tone, while the b* coordinate showed positive values, characteristic of yellow tones. The hue angle (°H) confirmed a predominant greenish-yellow colour, with the film remaining translucent.

### 2.7. Mechanical Properties

The mechanical properties of edible films are important to ensure that the film has adequate mechanical strength and integrity during transportation [75]. The mechanical properties evaluated on the films are shown in Table 5.

Fracture stress expresses the maximum stress developed in a film when subjected to an extension test, while the elongation value represents the extensibility of the film [76]. Chemical interaction between the components of the agroindustrial residue (cellulose, hemicellulose and lignin) with the grenetin, causing a greater binding force between them, with hydrophilic bonds, which has a direct impact on the property of resistance to rupture [77]. Elongation values reported in this study are higher than those reported by Niño et al. [77], for films made from lemon peel, polyvinyl alcohol, and sodium benzoate (3.02–5.9%), which can be attributed to the ability of gelatin to form gels that, together with its physicochemical properties, create synergy between the other components, allowing the formation of more flexible three-dimensional networks in biofilms [78].

#### Shelf Life Extension Assessment

Effect of biopolymeric coatings containing different carp hydrolysates on the shelf life of raspberries was evaluated through key physicochemical quality parameters over a 20-day storage period. The monitored variables included pH, titratable acidity, total soluble solids (°Brix), colour parameters (L*, a*, b*), and ascorbic acid content. Each parameter was plotted against storage time to visualize trends and compare effectiveness of the coatings.

**Figure 7:**
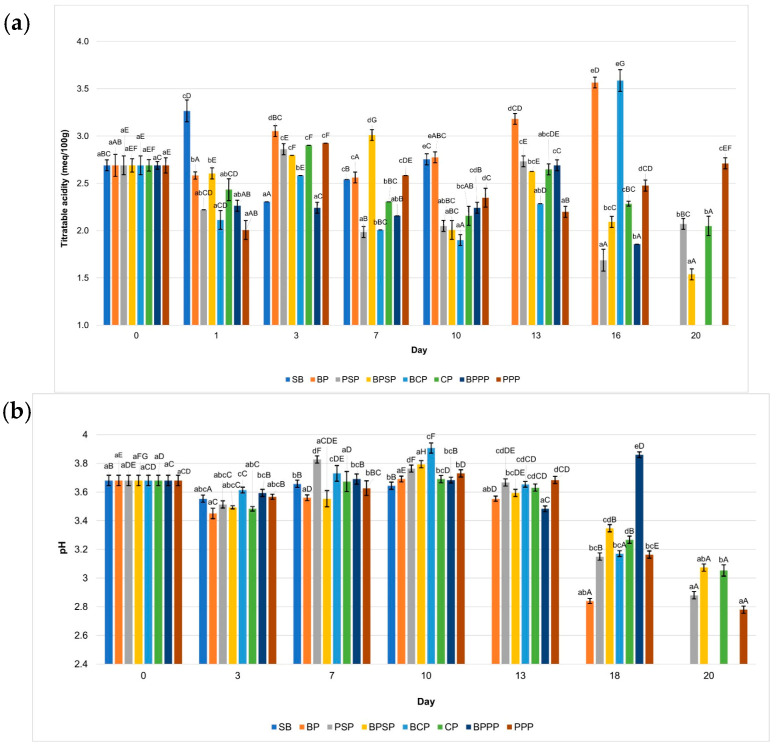
Changes in (**a**) titratable acidity and (**b**) pH during storage. Lowercase letters indicate significant differences among treatments on the same day, while uppercase letters indicate significant differences among storage times for the same treatment, according to ANOVA followed by Tukey’s HSD test (*p* < 0.05).

**Figure 8:**
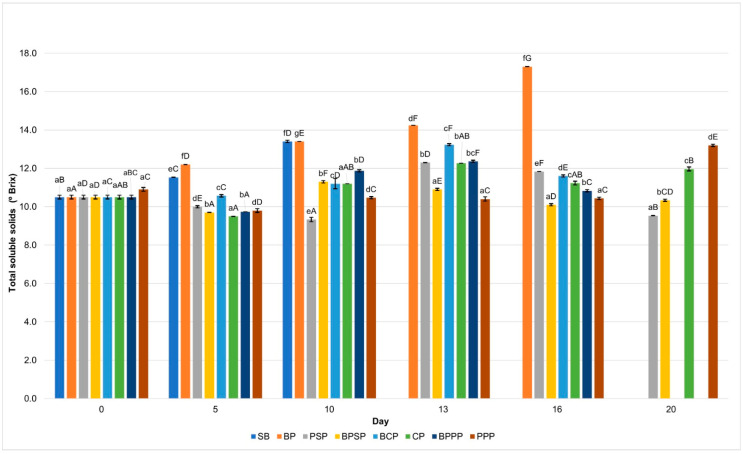
Total soluble solids. Lowercase letters, same day, significant differences between treatments and uppercase letters between time points for the same treatment, based on ANOVA and Tukey’s HSD test (*p* < 0.05).

**Figure 9:**
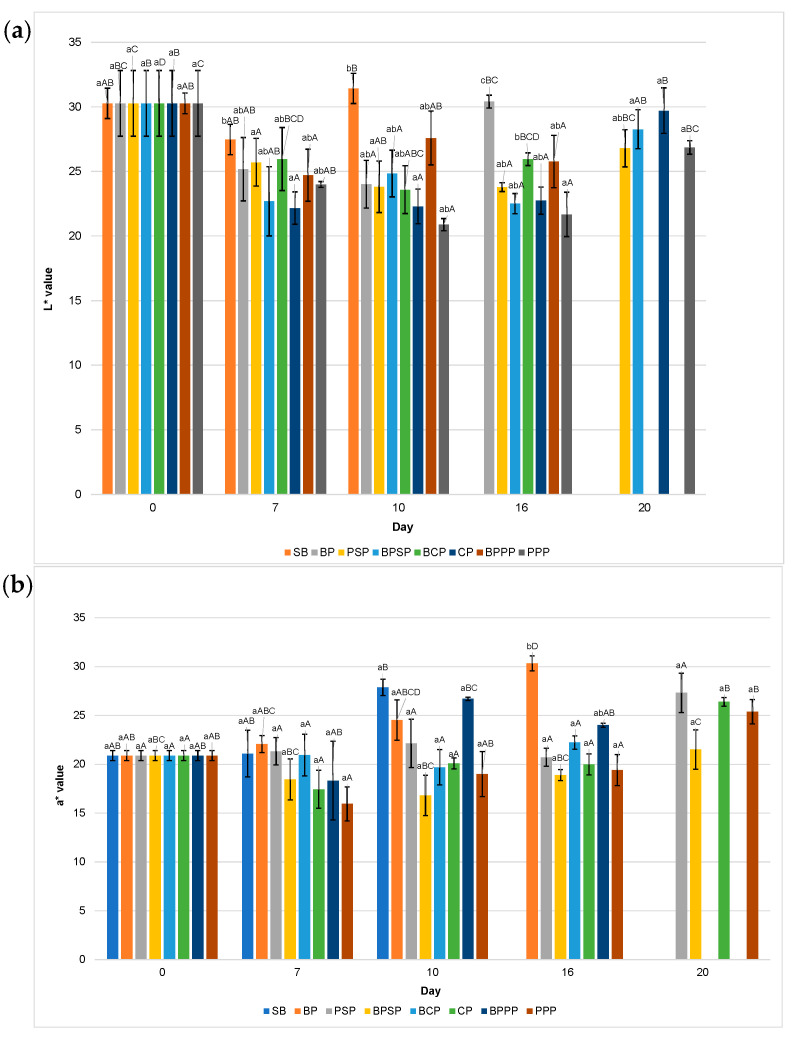

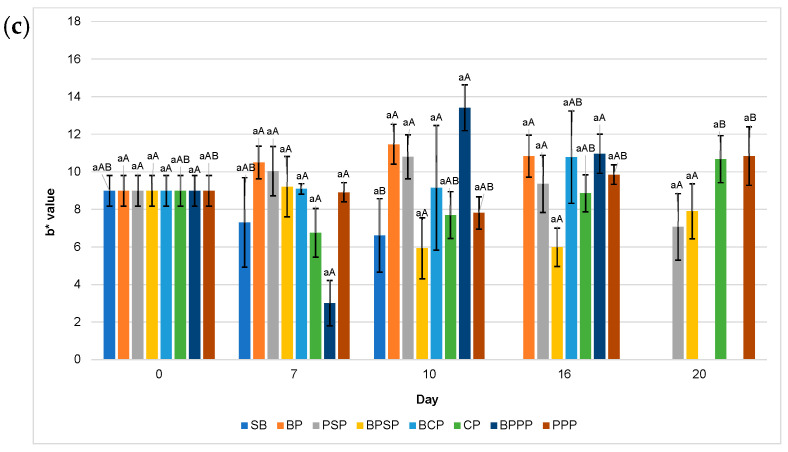
Colour: (**a**) L* values, (**b**) a* values, (**c**) b* values. Lowercase letters, same day, significant differences between treatments and uppercase letters between time points for the same treatment, based on ANOVA and Tukey’s HSD test (*p* < 0.05).

**Figure 10:**
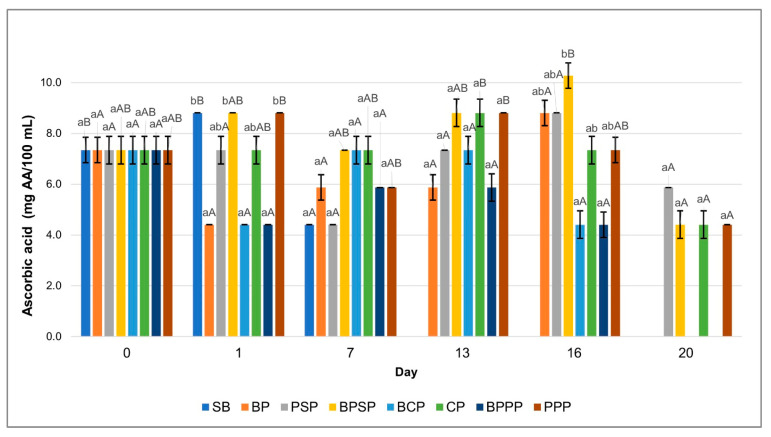
Ascorbic acid content. Lowercase letters, same day, significant differences between treatments and uppercase letters between time points for the same treatment, based on ANOVA and Tukey’s HSD test (*p* < 0.05).

To enhance the interpretation of the physicochemical evolution of raspberries during storage, the discussion highlights the key sampling days at which major changes or statistically significant shifts (*p* < 0.05) were observed across treatments. Based on the profiles shown in Figure 7, Figure 8, Figure 9 and Figure 10, the relevant time-points were identified as follows: Day 0, representing the initial condition of the fruit; Day 1, when early modifications in titratable acidity and ascorbic acid were first detected; Day 3, corresponding to the onset of clear divergences among coatings in pH, titratable acidity and colour parameters (particularly L* and a*); Days 5–7, marking a critical phase in which total soluble solids began to separate markedly among treatments, pH increased in uncoated controls, L* and a* exhibited their first significant declines, and ascorbic acid losses accelerated in coatings without hydrolysates; Day 10, when differences among treatments became more pronounced across all colour parameters and total soluble solids showed notable mid-storage increases in SB and BP; Day 13, indicating advanced deterioration in the controls, including significant reductions in pH, pigment stability and ascorbic acid content; Day 16, representing the point of maximum differentiation among treatments, particularly for total soluble solids (peak ripening in SB), a* values (greatest pigment degradation in SB and BP), and vitamin C retention (highest in PPP and PSP); and Day 20, the final stage of storage, where the cumulative effects on pH, titratable acidity, colour and ascorbic acid confirmed the contrasting preservation capacity of the tested coatings.

These sampling days were considered “key” because they coincided with: statistically significant differences among treatments, biochemically relevant turning points in ripening, oxidation or nutrient degradation, and clear performance divergence between hydrolysate-containing coatings (PPP, PSP) and controls (SB, BP).

Intermediate days that did not contribute meaningful transitions were not emphasised to maintain clarity and scientific relevance.

The results of this study demonstrate that the application of biopolymer coatings enriched with enzymatically obtained carp protein hydrolysates, particularly PPP treatment. Significantly enhances the preservation of key quality attributes in fresh raspberries during storage. The measured variables, including titratable acidity, pH, soluble solids, colour parameters, and ascorbic acid content, exhibited statistically significant differences (*p* < 0.05) both among treatments on the same day (denoted by lowercase letters) and within each treatment over time (denoted by uppercase letters), collectively supporting the conclusion that such coatings effectively extend raspberry shelf life.

### 2.8. Titratable Acidity and pH

Figure 7a shows that raspberries treated with PPP and PSP coatings maintained significantly lower pH (*p* < 0.05) values throughout storage period compared to uncoated control (SB) and other treatments, suggesting a slower ripening process and reduced microbial activity. Correspondingly, Figure 7b illustrates that titratable acidity was better preserved in the PPP and PSP treatments, while a marked decline was observed in the SB and BP samples. These findings support the hypothesis that protein hydrolysate based biopolymer coatings can effectively delay metabolic degradation and contribute to extended shelf life in raspberries.

PPP treatment showed a greater ability to maintain titratable acidity and prevent abrupt increases in pH during storage, compared to the control without coating (SB) and the biopolymer without hydrolysate (BP). This behavior suggests that PPP can delay ripening and limit microbial activity due to its ability to form a semi permeable barrier that reduces fruit respiration [79,80].

### 2.9. Total Soluble Solids

Figure 8 shows a progressive increase in TSS across all treatments, a trend typical of fruit ripening metabolism; however, PPP and PSP treatments exhibited significantly slower increases (*p* < 0.05). This suggests a moderating effect on respiratory and metabolic activity, likely resulting from the protective barrier formed by biopolymer coatings. In contrast, SB and BP controls showed more pronounced increases in TSS, which may be attributed to enhanced carbohydrate breakdown and accelerated ripening, consistent with findings reported by Romani et al. [81].

### 2.10. Colour (L*, a*, b*)

Figure 9 illustrates the evolution of colour parameters (L*, a*, b*) during storage, highlighting the superior performance of PPP and PSP treatments in preserving visual quality. Specifically, Figure 9a shows that fruit brightness (L*) was best maintained in raspberries coated with PPP and PSP, while a pronounced decline in lightness was observed in uncoated control (SB), likely due to surface dehydration or oxidative processes. Figure 9b reveals that red colour intensity (a*) decreased over time in all samples; however, coatings containing protein hydrolysates, particularly PPP significantly mitigated this loss compared to SB, BP, and buffer only controls. Similarly, Figure 9c displays greater stability in the yellow-blue tonality (b*) in PPP and PSP treated raspberries, whereas SB group exhibited pronounced colour degradation. These findings suggest that coatings enriched with enzymatically obtained protein hydrolysates offer enhanced protection against pigment oxidation, consistent with previous reports on efficacy of biopolymer based edible coatings [80].

### 2.11. Changes in Ascorbic Acid Content During Storage

Figure 10 shows a significant decline (*p* < 0.05) in ascorbic acid content over the storage period across all treatments; however, coatings containing protein hydrolysates, particularly PPP and PSP were more effective in mitigating this degradation. The enhanced retention of ascorbic acid in these treatments is likely attributed to the presence of bioactive peptides with antioxidant properties in the hydrolysates, which help reduce oxidative loss of the compound. These findings are consistent with previous studies indicating that protein hydrolysate based coatings can provide functional protection for labile nutrients such as vitamin C [79,81].

PPP was the most effective treatment in terms of overall preservation of physicochemical parameters. PSP also performed well, although slightly less so. CP performed moderately. The controls with buffer only (BPPP, BPSP, BCP) and BP were less effective, and the SB treatment was the most susceptible to deterioration.

Biopolymer coatings with carp hydrolysates, especially those made with bromelain (PPP), represent a promising alternative for extending the shelf life of fresh raspberries by preserving their physicochemical quality. These findings are in line with previous studies reporting beneficial effects of protein hydrolysates on the preservation of fruits and vegetables [82,83].

## 3. Discussion

The present study demonstrates that hybrid biopolymers formulated from agri-food and fishery by-products, enriched with enzymatically generated carp protein hydrolysates, constitute an effective strategy for extending the shelf life of raspberries within a circular bioeconomy framework. The coating based on fish gelatin, whey protein, orange peel fiber, and glycerol, incorporating hydrolysates obtained with papain- and bromelain-rich preparations, combined adequate mechanical performance and controlled solubility with relevant antioxidant functionality, resulting in improved preservation of pH, titratable acidity, colour, and ascorbic acid content during refrigerated storage.

Coatings containing the bromelain-derived hydrolysate (PPP) provided the most pronounced preservation of physicochemical quality, even though PSP hydrolysates exhibited slightly higher radical scavenging capacity in bulk DPPH^•^ and ABTS^•+^ assays. This apparent discrepancy between in vitro antioxidant rankings and in situ coating performance can be explained by differences in peptide profile, hydrophobicity, and peptide–polymer interactions. Bromelain typically generates shorter, more hydrophobic, and aromatic-residue-rich peptides than papain, favouring their localisation at the coating–fruit interface and within less polar microdomains of the gelatin–whey–citrus fibre network [84]. Under these conditions, PPP-derived peptides can more efficiently scavenge radicals, chelate transition metals, and stabilise anthocyanins and ascorbic acid at the fruit surface, where oxidative reactions are most intense. In contrast, the more polar peptide population produced by papain may display high antioxidant capacity in solution but interact differently with the polymer matrix, limiting its interfacial efficacy. This structure–function behaviour is consistent with reports showing that low-molecular-weight, hydrophobic marine-derived peptides enhance oxidative stability and quality retention when incorporated into protein–polysaccharide films and coatings [20,85,86].

The combined FTIR and DSC analyses further support these mechanistic interpretations. PPP and PSP hydrolysates exhibited greater amide-band depletion and more defined high-temperature transitions than pepsin hydrolysates and non-enzymatic controls, indicating a higher proportion of short, mobile peptide chains with altered hydrophobic interactions. These structural features correlate with the superior radical-scavenging and reducing power observed for enzymatic hydrolysates, particularly P+PSP and P+PPP, and help explain their capacity to modulate oxidation-driven changes in colour and vitamin C during storage. The ranking PPP > PSP > CP > P-E in terms of hydrolysis degree and thermal behaviour is coherent with the hierarchy observed for functional performance in raspberries, reinforcing the link between peptide structure, matrix interactions, and preservation outcomes.

From the materials science perspective, the casein–gelatin–citrus fibre biopolymer displayed moisture content, water holding capacity, solubility and mechanical properties within the ranges reported for edible films designed for fresh-fruit application [19,69,72,73,74]. The film exhibited sufficient tensile strength and extensibility to withstand handling, together with a solubility of approximately 50%, which favours gradual release of antioxidant peptides while avoiding excessive loss of structural integrity. The viscosity of the film-forming solution enabled uniform coating by dipping, and the colour parameters (high L*, slight greenish–yellow hue) were compatible with the visual acceptance of raspberries. These characteristics align with recent advances in protein–polysaccharide films and composite systems aimed at functional packaging and fruit preservation [20,69,70,71].

Beyond laboratory performance, the formulation possesses several attributes that facilitate its potential scale-up. The rheological behaviour of the film-forming dispersion is compatible with immersion, spraying, and continuous casting processes, and the polymeric matrix tolerates typical industrial temperature ranges without critical degradation, as indicated by DSC. The exclusive use of food-grade, edible components simplifies regulatory approval and reduces the need for complex migration or toxicity studies, in agreement with current trends in biopolymer-based food packaging [87,88]. In addition, the reliance on locally available by-products—citrus peels, carp processing residues, and plant-derived enzymes—enhances environmental and economic sustainability, offering opportunities for decentralised implementation in regions with high agro-industrial waste generation.

Overall, the peptide-enriched biopolymer developed here can be regarded as a technically feasible and environmentally sound candidate within the new generation of functional edible coatings for postharvest preservation. The results highlight the importance of tailoring enzymatic hydrolysis conditions and peptide profiles to maximize interfacial antioxidant efficacy rather than relying solely on bulk in vitro assays. Future research should address a more detailed molecular characterization of the peptide fractions (LC–MS/MS profiling and fractionation), microbial shelf-life assessment, sensory evaluation, and pilot-scale trials under realistic packing and cold-chain conditions. Life cycle assessment and techno-economic analysis will also be necessary to support technology transfer and integration into commercial fresh-fruit preservation systems.

## 4. Materials and Methods

### 4.1. Chemical Reagents

Pepsin from porcine gastric mucosa, DPPH^•^ (1,1-diphenyl-2-picrylhydrazyl), ABTS^•+^ (2,2′-azino-bis(3-ethylbenzothiazoline-6-sulfonic acid)), Trolox (6-hydroxy-2,3,7,8-tetramethylchroman-2-carboxylic acid), potassium ferricyanide, trichloroacetic acid (TCA), and ferric chloride were obtained from Sigma-Aldrich (Mexico City, Mexico). All solvents were of analytical grade. Phosphate and citrate buffers were purchased from Sigma-Aldrich and used as received.

### 4.2. Enzymatic Preparations

Pineapple peels were collected from minimal-processing waste (Atlacomulco, Mexico), washed, and homogenized using a Turmix extractor (Rudo model; Turmix S.A. de C.V., Mexico City, Mexico). PPP were obtained following Gallardo et al. [89], with minor modifications. Briefly, 100 g of peels were mixed with 150 mL ethanol and stored at −10 °C for 7 days. The ethanol–extract mixture was centrifuged at 3000× *g* for 20 min at 4 °C, yielding a yellow precipitate that was dried at room temperature and stored in opaque containers.

The pH of the pineapple peel enzymatic extract was 7.3, consistent with the alkaline conditions typically associated with bromelain-rich preparations.

Papaya seeds were collected from processing waste (Atlacomulco, Mexico), washed, dried at 60 °C for 4 h, ground, and sieved through a No. 100 mesh (149 μm), according to Galindo-Estrella et al. [90]. The powdered material was stored in dark containers until use. The pH of the papaya seed extract was 6.0, within the optimal range reported for papain-containing preparations.

#### 4.2.1. Proteolytic Activity of Enzymatic Preparations

Proteolytic activity was determined according to the Kunitz method [91] using 1% (*w*/*v*) casein as substrate. Enzyme solutions were incubated with the casein suspension in 50 mM phosphate buffer (pH 7.5) at 37 °C for the specified reaction time. The reaction was stopped by adding trichloroacetic acid (TCA), which precipitated undigested proteins. Samples were then filtered or centrifuged, and the absorbance of the supernatant was measured at 280 nm. One unit of protease activity was defined as the amount of enzyme required to release 1 µg of tyrosine per minute under the assay conditions.

#### 4.2.2. Electrophoresis of Enzymatic Preparations

SDS-PAGE (Sodium dodecyl sulfate polyacrylamide gel electrophoresis) was carried out according to the methodology described by López-Medina et al. [92]. The analysis was carried out under a constant current of 200 volts, employing a Mini-Protean II Slab Cell electrophoresis instrument from Bio-Rad (Richmond, CA, USA) at 4° C. A volume of 7 µL was used for the samples and 5 µL for the molecular weight marker.

### 4.3. Acquisition of Common Carp Subproduct Hydrolysates

#### 4.3.1. Substrate

The waste of *Cyprinus carpio* (skin, head, bones and fins) were obtained from San Luis Mextepec Market, Zinacantepec, Toluca, Mexico; 10 kg of subproducts were washed with abundant tap water and heated with purified water, to boiling point, for 10 min. Excess water was eliminated, and the sample was dried at 45 °C for 12 h. The dried material was then ground using a Ninja blender (model BN801) and sieved through a No. 80 (180 µm) mesh to obtain a uniform particle size. The processed material was stored in a dark container until further use.

#### 4.3.2. Enzymatic Hydrolysis

The hydrolysis reactions for each enzymatic preparation were carried out according to the methodology described by Jae et al. [93], with modifications. A 1:4 (*m*/*v*) ratio of common carp by-product protein was used as substrate, phosphate buffer 0.1 M (pH 7.3) for the common carp protein + pineapple peel enzymatic preparation (P+PPP), pH 6.0 for the common carp protein + papaya seed enzymatic preparation (P+PSP) and citrate buffer 0.1 M pH 2.0 buffer for the protein with commercial pepsin (P+CP) as a positive control, and carp protein in absence of enzymes (P-E) was used as a blank control. 1 g of enzymatic preparations were added to the flour of the carp by-products and were incubated for 8 h.

After the incubation period, the enzymes were inactivated at 95° C for 10 min. Afterwards, the mixture was rapidly cooled and centrifuged at 10,000× *g* for 20 min at 4° C. The supernatant obtained was lyophilized and stored in a dark container until further analysis.

### 4.4. Identification of Hydrolysates by FTIR and In Silico Analysis

The FTIR (Fourier Transform Infrared Spectroscopy) spectrum was obtained using a Jasco FT/IR 400 spectrometer (Jasco, Tokyo, Japan) over a wavenumber range of 600–4000 cm^−1^, with a resolution of 8 cm^−1^ and 26 scans.

In silico research was carried out in the databases cited in Table 6, using the methodology reported by López-García et al. [94].

### 4.5. Thermal Analysis

The thermal behavior of the hydrolysates was evaluated using a NETZSCH DSC 204 F1 Phoenix^®^ thermal analyzer (NETZSCH-Gerätebau GmbH, Selb, Germany). Differential scanning calorimetry (DSC) was performed using approximately 5 mg of sample placed in alumina crucibles and heated from 25 °C to 380 °C at a rate of 10 °C/min under a nitrogen flow of 20 mL/min. A total of 26 scans were recorded for each hydrolysate. All thermal analyses were performed in duplicate for each hydrolysate preparation.

### 4.6. Soluble Protein Content

The determination of soluble protein was carried out via the biuret method [95]. The samples were read at 540 nm using a UV-Vis spectrophotometer (Velaquin, model VE-5600UV, Mexico City, Mexico). The standard curve was obtained via bovine serum albumin at concentrations ranging from 0–10 mg/mL.

### 4.7. Antioxidant Activity

#### 4.7.1. ABTS^•+^ (2,2′-Azino-bis 3-Ethylenebenzothiazoline-6-sulfonic Acid)

The method described by Alam et al. [96] was employed for the determination of antioxidant activity. To 1 mL of the ABTS reagent solution, 100 µL of 2 mM Trolox (positive control), methanol (blank) or hydrolysates were added. The absorbance reading was acquired 10 min after combining the extract with the ABTS^•+^ reagent solution (7 mM) at 734 nm.

#### 4.7.2. DPPH^•^ (2,2-Diphenyl-1-picrylhydrazyl)

The antiradical activity determination method was adapted from Alam et al. [96]. The decrease in the absorbance of DPPH^•^ was measured at 520 nm, reacting of 2.8 mL of DPPH^•^ (0.1 mM) and 100 µL of 2 mM Trolox (positive control), methanol (blank) or hydrolysates.

#### 4.7.3. Reducing Power (RP)

The procedure outlined by Alam et al. [96] was followed. To 1.0 mL of the sample dissolved in distilled water, 2.5 mL of 0.2 M phosphate buffer (pH 6.6) and 2.5 mL of K_3_Fe(CN)_6_ (1% *w*/*v*) were added. The mixture was incubated at 50 °C for 20 min, after which 2.5 mL of TCA (10% *w*/*v*) was added. The solution was then centrifuged at 3000× *g* for 10 min to obtain the upper layer (2.5 mL), which was mixed with 2.5 mL of distilled water and 0.5 mL of FeCl_3_ (0.1% *w*/*v*). Absorbance was measured at 700 nm, using a 2 mM glutathione solution as a positive antioxidant control.

### 4.8. Obtaining Raw Materials Based on Agrifood By-Products for Biopolymers

#### 4.8.1. Obtaining and Characterization of Agrifood By-Product Fiber (Orange Peel)

To obtain orange fiber, peels of *Citrus sinensis* were collected from minimal process food from Atlacomulco, Mexico Market; the process followed the method of Avena-Bustillos et al. [61]. Products proximate composition was determined, identifying its main functional groups by FTIR and stored in polyethylene bags in a desiccator until its further use [97].

#### 4.8.2. Obtaining and Characterization of Grenetin from Fish By-Products

Fish by-products (skin, head, bones and fins) from San Luis Mextepec Market, Zinacantepec, Toluca, Mexico, were used to obtain grenetin according to Avena-Bustillos et al. [61]. Grenetin was characterized by FTIR and according to NORMEX [66], dried and stored in polyethylene bags in a desiccator until posterior analysis.

#### 4.8.3. Obtaining and Characterization of Whey Protein Isolate

Whey protein isolate was obtained by precipitation of whey from cheese production at the dairy plant, Faculty of Chemistry, UAEMex. It was characterized by FTIR and according to NOM-243-SSA1-2010 [62]. The material obtained was dried and stored in polyethylene bags in a desiccator until further use.

### 4.9. Biopolymer Development

Biopolymer development was conducted following the procedure described by García-Argueta et al. [78]. Carp protein hydrolysates were incorporated at 3% (*w*/*w*). This concentration ensured uniform dispersion within the matrix, prevented phase separation, and maintained the structural cohesion of the film-forming solution. The pH of the film-forming solution was 6.3 ± 0.1. The selected level is consistent with ranges commonly used for peptide-enriched edible coatings, where 1–5% additions have been reported to enhance antioxidant functionality while preserving the mechanical integrity of protein–polysaccharide films [19,20,86].

#### Physical and Mechanical Properties

For the characterization of the biopolymer, the following analyses were carried out: thickness, measured with a digital micrometer following Valdez-Valdez et al. [58]; moisture content according to AOAC [98]; water holding capacity following ASTM D5229/D5229M-92 [99]; solubility based on a modification of Gómez-Estaca et al. [100]; and colour measured using a Konica Minolta Chroma Meter CR-400 (Sensing Inc., Tokyo, Japan) calibrated with plate No. 12633047 and standard values L = 97.3, a = 0.17, and b = 1.9, as reported by López-Medina et al. [101].

The viscosity of the film-forming solution was measured prior to coating using a Brookfield Viscometer (Model RVDV-I, Brookfield Engineering Labs Inc., Middleboro, MA, USA) equipped with a No. 2 spindle at 60 rpm and 25 °C. Measurements were performed in triplicate and expressed in mPa·s.

**Figure 11:**
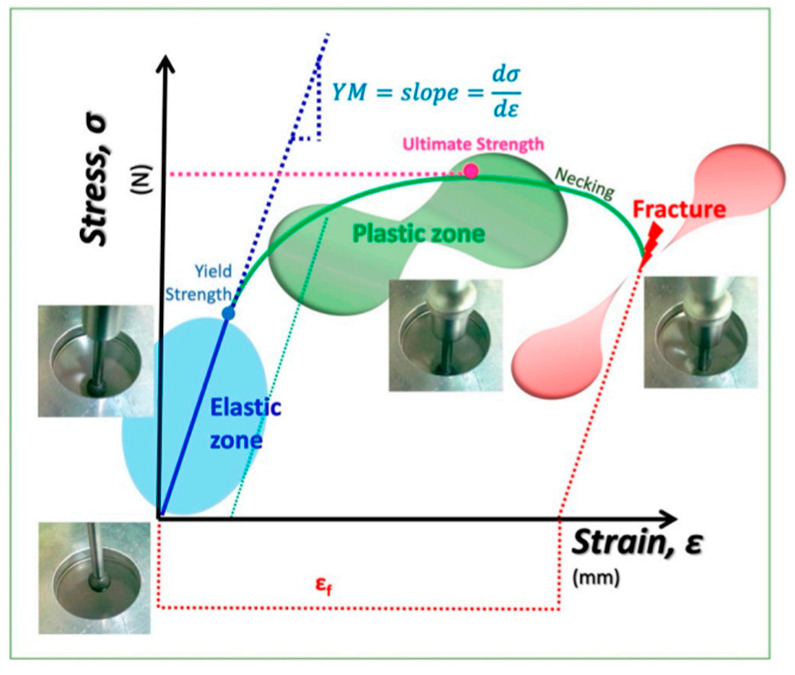
Schematic stress–strain curve illustrating the elastic, plastic, and fracture regions typical of film mechanical testing [74] Herrera-Vázquez et al.

The mechanical properties of the biopolymer films, breaking strength, elasticity, percentage elongation (% E), and Young’s modulus (YM), were determined using a TA-XT2 Texture Analyzer (Stable Micro Systems Ltd., Goadalming, Surrey, UK), following the methodology described by García-Argueta et al. [78] with minor modifications. A stainless-steel spherical probe (0.255 1/4″) was operated at a test speed of 1.0 mm/s and a penetration distance of 30.0 mm to obtain the force–deformation curves (Figure 11).

The mechanical parameters determined for the biopolymer highlight its structural integrity and flexibility, both of which are essential to ensuring mechanical stability during handling and transport [75].

### 4.10. Application of Biopolymer Coating to Raspberries

Raspberries used in the shelf-life study were obtained from the San Martín supply center (Atlacomulco, State of Mexico) at commercial ripeness, ensuring uniform colour, size, and absence of skin damage. Fruits were washed and disinfected with a 0.05% (*v*/*v*) sodium hypochlorite solution, rinsed with distilled water, and air-dried at room temperature for 10 min. Each treatment (SB, BP, BPPP, BPSP, BCP, PPP, PSP, CP) was applied to 90 raspberries, distributed into three independent biological replicates of 30 fruits each.

The fruits were coated by immersion in the corresponding biopolymer solution for 20 s, drained, and dried at room temperature. Coated raspberries were stored in ventilated plastic containers at 4 °C until analysis. For each sampling day, ten fruits per replicate (*n* = 30 per treatment) were randomly selected for physicochemical evaluations, following validated methodologies for berry coating studies [19,81].

### 4.11. Physicochemical Characteristics of Coated Raspberries Stored Under Refrigeration

Physicochemical parameters, colour, total soluble solids (TSS), pH, ascorbic acid content (AA), and titratable acidity of the coated raspberries stored at 4 ± 1 °C were evaluated every two days for a period of 20 days. All measurements were performed in triplicate until evident microbiological deterioration (presence of fungal hyphae) was observed.

#### 4.11.1. Colour Measurement

External colour of the blackberries was measured using a Konica Minolta colourimeter (model Chroma Meter CR-400, Sesing, Inc., Tokyo, Japan), with calibration plate number 12633047 and standards of L = 97.3, a = 0.17, b = 1.9. Samples were longitudinally sliced, and direct readings were taken from external surfaces in triplicate for the CIELAB colour space coordinates: L* (lightness), a* (red/green), b* (yellow/blue) [101].

#### 4.11.2. pH and Titratable Acidity

pH was quantified with a Thermo Scientific potentiometer, model Orion 720-A, following the methodology of [102].

Titratable acidity was determined by a volumetric acid-base titration method, using 0.1 N sodium hydroxide (NaOH) as titration agent and phenolphthalein as indicator. The results were expressed as meq of citric acid/100 mg. This analysis was performed according to the methodology described in AOAC methods [103].

#### 4.11.3. Total Soluble Solids (°Brix)

TSS (°Brix) was determined according to NMX-F-112-NORMEX-2010 [104]. For this purpose, the juice was extracted from the sample by pressure and subsequently measured using a digital refractometer (ATAGO^®^ Pocket, Tokyo, Japan) with automatic temperature compensation, with a measuring scale from 0 to 90%.

#### 4.11.4. Ascorbic Acid Content

Ascorbic acid content (AA) was determined by a volumetric titration method, using 0.05 N iodine as titration agent and starch as indicator. The results were expressed in mg AA/mL. The procedure was carried out in accordance with NOM-243-SSA1-2010 [62].

## 5. Conclusions

This study demonstrates that biopolymer coatings formulated from citrus fiber, fish gelatin, whey protein, and glycerol, and enriched with carp protein hydrolysates, effectively preserved the physicochemical quality of raspberries during refrigerated storage. The superior performance of the bromelain-derived hydrolysate (PPP) is attributed to its short, hydrophobic, and aromatic-rich peptide profile, which favors stronger interactions with the gelatin–whey–fiber matrix and enhances interfacial antioxidant activity. These mechanisms explain the improved stabilization of anthocyanins, ascorbic acid, and color parameters compared with coatings lacking hydrolysates.

The DSC data confirmed the thermal stability of the enzymatic hydrolysates up to 90 °C, supporting their suitability for cold-chain applications and aligning with recent reports on thermally stable bioactive peptides for active packaging systems. The functional hierarchy observed (PPP > PSP > CP > P–E) closely reflects differences in peptide structure and mobility, reinforcing the link between molecular properties and preservation outcomes.

Overall, incorporating carp protein hydrolysates into biodegradable matrices represents a feasible and sustainable strategy with which to enhance the shelf life of perishable fruits. Future work should focus on peptide profiling (LC–MS/MS), microbial stability, and pilot-scale validation to support the translation of this technology into commercial edible coating applications.

## Figures and Tables

**Table 1:** Specific activity of enzyme preparations.

Sample	Specific Activity (U/mg Protein)
PPP	969.4 ± 58.5 ^b^
PSP	420.2 ± 7.4 ^a^
CP	430.6 ± 15.9 ^a^

Different letters indicate statistically significant differences *p* < 0.05.

**Table 2:** Soluble protein and antioxidant activity of protein hydrolysates prepared from common carp (*Cyprinus carpio*) waste.

Sample	IC_50_ ABTS^•+^ (mg Hydrolysate/mL)	IC_50_ DPPH^•^ (mg Hydrolysate/mL)	Soluble Protein (mg/mL)
P-E	1.1 ± 0.1 ^c^	71.3 ± 1.0 ^b^	3.6 ± 0.2 ^a^
P+PPP	1.3 ± 0.1 ^b^	58.1 ± 0.5 ^c^	3.1 ± 0.1 ^b^
P+PSP	0.8 ± 0.1 ^d^	31.6 ± 0.3 ^d^	2.4 ± 0.1 ^c^
P+CP	1.7 ± 0.1 ^a^	76.7 ± 0.9 ^a^	3.1 ± 0.1 ^b^

Different letters indicate statistically significant differences. P-E (Carp protein in absence of enzymes); P+PPP (Common carp protein + pineapple peel enzymatic preparation); P+PSP (Common carp protein + papaya seed enzymatic preparation); P+CP (Common carp protein with the added commercial pepsin).

**Table 4:** Average values of the physical properties of the biopolymer film.

Parameter	Results
Thickness (µm)	0.17 ± 0.05
Moisture (%)	33.80 ± 1.24
WHC (%)	161.57 ± 3.30
S (%)	49.78 ± 4.75
L*	91.22 ± 0.31
a*	−2.10 ± 0.49
b*	12.16 ± 2.20
C	12.51 ± 2.14
°H	100.02 ± 0.16
ΔE	12.50 ± 2.04
Viscosity (mPa·s)	143.96 ± 4.78

Results correspond to triplicate measurements of the single optimized formulation developed in this study.

**Table 5:** Mechanical properties of biopolymer based on agri-food by-products.

Parameter	Results
Resistance (N)	5.03 ± 1.87
Elongation (mm)	19.22 ± 0.73
% of elongation	51.31 ± 5.71
YM (N/mm)	0.32 ± 0.12

**Table 6:** Biopeptide databases.

Database	Web Address	Content
AHTPDB *	https://biochemia.uwm.edu.pl/biopep/start_biopep.php /(accessed on 14 December 2025)	Antihypertensive peptides
AntiTbPdb	http://webs.iiitd.edu.in/raghava/antitbpdb (accessed on 14 December 2025)	Antitubercular and mycobacterial peptides
APD	http://aps.unmc.edu/AP/ (accessed on 14 December 2025)	Antimicrobial and anticancer peptides
AVPdb	http://crdd.osdd.net/servers/avpdb/ (accessed on 14 December 2025)	Antiviral peptides
BaAMPs	http://www.baamps.it/ (accessed on 14 December 2025)	Antimicrobial peptides tested against microbial films
BactPepDB	http://bactpepdb.rpbs.univ-paris-diderot.fr/cgi-bin/home.pl (accessed on 14 December 2025)	Bacterial peptides
BIOPEP-UWMTM *	http://www.uwm.edu.pl/biochemia (accessed on 14 December 2025)	Bioactive peptides/sensory peptides and amino acids
Brainpeps	http://brainpeps.ugent.be/ (accessed on 14 December 2025)	Blood-brain barrier passing peptides
CAMP_R3_	https://camp.bicnirrh.res.in/ (accessed on 14 December 2025)	Antimicrobial peptides
CancerPPD	http://crdd.osdd.net/raghava/cancerppd/index.php (accessed on 14 December 2025)	Anticancer peptides and proteins
CPPSite 2.0	http://crdd.osdd.net/raghava/cppsite/ (accessed on 14 December 2025)	Cell-penetrating peptides
DBAASP	https://dbaasp.org/ (accessed on 14 December 2025)	Antimicrobial peptides
EROP-Moscow	https://academic.oup.com/nar/article/34/suppl_1/D261/1132217 / (accessed on 14 December 2025)	Bioactive peptides
Hemolytik	http://crdd.osdd.net/raghava/hemolytik/ (accessed on 14 December 2025)	Hemolytic and non-hemolytic peptides
MBPDB *	http://mbpdb.nws.oregonstate.edu/ (accessed on 14 December 2025)	Milk protein-derived bioactive peptides
NeuroPep	http://isyslab.info/NeuroPep/ (accessed on 14 December 2025)	Neuropeptides
PepBank	https://pubmed.ncbi.nlm.nih.gov/17678535/ (accessed on 14 December 2025)	Bioactive peptides
Quorumpeps	http://quorumpeps.ugent.be/ (accessed on 14 December 2025)	Quorum sensing signaling peptides
SATPdb	http://crdd.osdd.net/raghava/satpdb/links.php (accessed on 14 December 2025)	A metabase of therapeutic peptides
StraPep	http://isyslab.info/StraPep/ (accessed on 14 December 2025)	Structures of bioactive peptides
THPdb	http://crdd.osdd.net/raghava/thpdb/index.html (accessed on 14 December 2025)	FDA-approved therapeutic peptides
TumorHoPe	http://crdd.osdd.net/raghava/tumorhope/ (accessed on 14 December 2025)	Tumor homing peptides
YADAMP	http://yadamp.unisa.it/about.aspx (accessed on 14 December 2025)	Antimicrobial peptides

Databases marked with an asterisk (*) are widely used and curated databases frequently employed for bioactive peptide identification and analysis.

## Data Availability

The original contributions presented in this study are included in the article. Further inquiries can be directed to the corresponding author.

## Abbreviations

The following abbreviations are used in this manuscript:

AA	Ascorbic acid content
ABTS^•+^	2,2′-Azino-bis 3-ethylenebenzothiazoline-6-sulfonic acid
BOD	Biochemical oxygen demand
CP	Commercial pepsin
DSC	Differential Scanning Calorimetry
DPPH^•^	1,1-diphenyl-2-picrylhydrazyl
% E	Percentage elongation
FRAP	Ferric Reducing Antioxidant Power
FTIR	Fourier Transform Infrared Spectroscopy
GSH,	glutathione standard
IC_50_	Mean inhibitory concentration
MY	Young’s modulus
P+CP	Common carp protein with the added commercial pepsin
P-E	Carp protein in absence of enzymes
PPP	Pineapple peel preparations
P+PPP	Common carp protein + pineapple peel enzymatic preparation
PSP	Papaya seed preparations
P+PSP	Common carp protein + papaya seed enzymatic preparation
RP	Reducing power
SB	Blanck control
SDS-PAGE	Sodium dodecyl sulfate polyacrylamide gel electrophoresis
TCA	Trichloroacetic acid
TSS	Total soluble solids
Trolox	6-hydroxy-2,3,7,8-tetramethylchroman-2-carboxylic acid
WRC	Water holding capacity
